# Exploring the role of anticipatory postural adjustment duration within APA2 subphase as a potential mediator between clinical disease severity and fall risk in Parkinson’s disease

**DOI:** 10.3389/fnagi.2024.1354387

**Published:** 2024-06-25

**Authors:** Cheng-Hao Hu, Yun-Ru Lai, Chih-Cheng Huang, Chia-Yi Lien, Yueh-Sheng Chen, Chiun-Chieh Yu, Sieh-Yang Lee, Wei-Che Lin, Ben-Chung Cheng, Wen-Chan Chiu, Yi-Fang Chiang, Chien-Feng Kung, Cheng-Hsien Lu

**Affiliations:** ^1^Department of Neurology, Kaohsiung Chang Gung Memorial Hospital, Chang Gung University College of Medicine, Kaohsiung, Taiwan; ^2^Department of Hyperbaric Oxygen Therapy Center, Kaohsiung Chang Gung Memorial Hospital, Chang Gung University College of Medicine, Kaohsiung, Taiwan; ^3^Department of Radiology, Kaohsiung Chang Gung Memorial Hospital, Chang Gung University College of Medicine, Kaohsiung, Taiwan; ^4^Department of Radiology, Kaohsiung Chang Gung Memorial Hospital, Chang Gung University College of Medicine, Kaohsiung, Taiwan; ^5^Department of Internal Medicine, Kaohsiung Chang Gung Memorial Hospital, Chang Gung University College of Medicine, Kaohsiung, Taiwan; ^6^Department of Intelligent Commerce, National Kaohsiung University of Science and Technology, Kaohsiung, Taiwan; ^7^Department of Biological Science, National Sun Yat-Sen University, Kaohsiung, Taiwan; ^8^Department of Neurology, Xiamen Chang Gung Memorial Hospital, Xiamen, Fujian, China

**Keywords:** Parkinson’s disease, fall risk, Tinetti balance and gait score, anticipatory postural adjustments, center of pressure trajectories, Unified Parkinson’s Disease Rating Scale

## Abstract

**Introduction:**

People with Parkinson’s Disease (PD) often show reduced anticipatory postural adjustments (APAs) before voluntary steps, impacting their stability. The specific subphase within the APA stage contributing significantly to fall risk remains unclear.

**Methods:**

We analyzed center of pressure (CoP) trajectory parameters, including duration, length, and velocity, throughout gait initiation. This examination encompassed both the postural phase, referred to as anticipatory postural adjustment (APA) (APA1, APA2a, APA2b), and the subsequent locomotor phases (LOC). Participants were instructed to initiate a step and then stop (initiating a single step). Furthermore, we conducted assessments of clinical disease severity using the Unified Parkinson’s Disease Rating Scale (UPDRS) and evaluated fall risk using Tinetti gait and balance scores during off-medication periods.

**Results:**

Freezing of gait (FOG) was observed in 18 out of 110 participants during the measurement of CoP trajectories. The Ramer-Douglas-Peucker algorithm successfully identified CoP displacement trajectories in 105 participants (95.5%), while the remaining 5 cases could not be identified due to FOG. Tinetti balance and gait score showed significant associations with levodopa equivalent daily dose, UPDRS total score, disease duration, duration (s) in APA2a (s) and LOC (s), length in APA1 (cm) and APA2b (cm), mediolateral velocity in APA1 (X) (cm/s), APA2a (X) (cm/s), APA2b (X) (cm/s) and LOC (X) (cm/s), and anterior–posterior velocity in APA2a (Z) (cm/s) and APA2b (Z) (cm/s). Multiple linear regression revealed that only duration (s) in APA2a and UPDRS total score was independently associated with Tinetti gait and balance score. Further mediation analysis showed that the duration (s) in APA2a served as a mediator between UPDRS total score and Tinetti balance and gait score (Sobel test, *p* = 0.047).

**Conclusion:**

APA2 subphase duration mediates the link between disease severity and fall risk in PD, suggesting that longer APA2a duration may indicate reduced control during gait initiation, thereby increasing fall risk.

## Introduction

Inefficient weight shifts are a prevalent factor leading to falls in individuals with Parkinson’s Disease (PD) (Tard et al., 2013; Lira et al., 2020; Russo et al., 2022). During quiet standing, the body’s center of mass (CoM) aligns vertically with the center of pressure (CoP). However, a dissociation between the CoP and CoM that occurs after stepping, known as CoM-CoP decoupling, results in an unstable stance leg condition (Winter et al., 1990; Plate et al., 2016).

**Figure 1:**
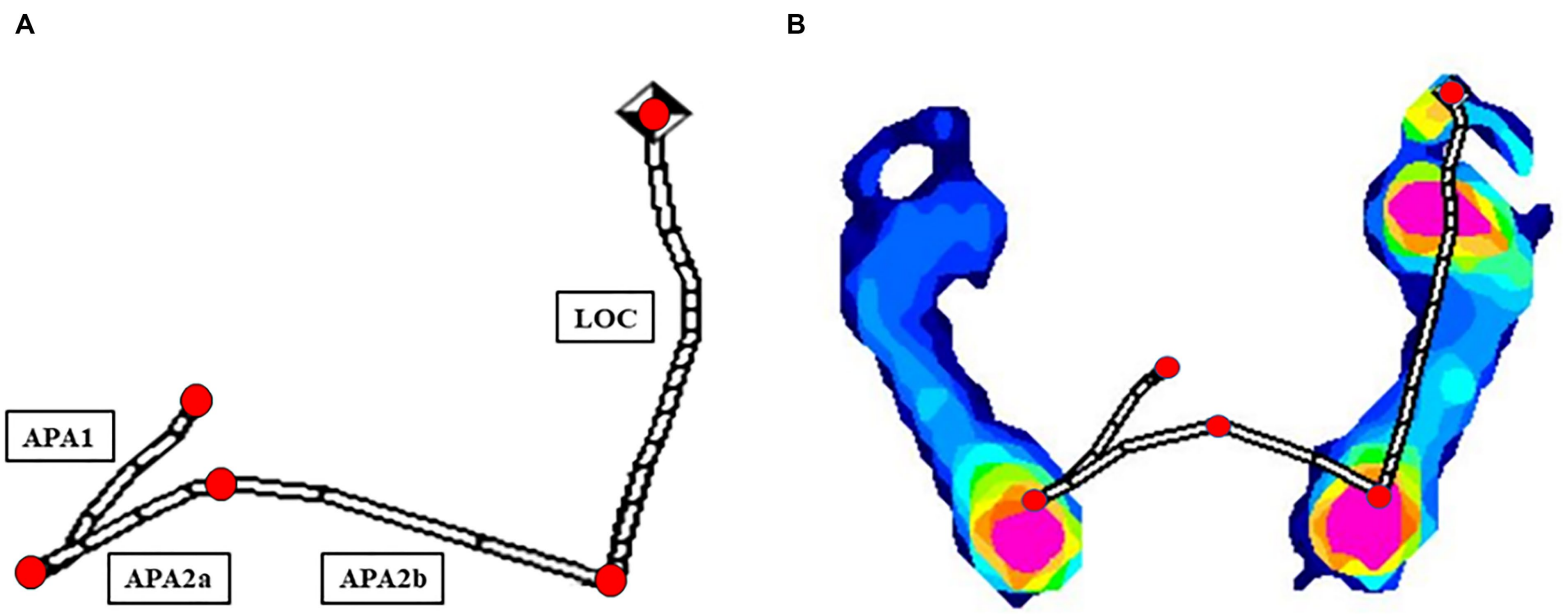
Computational analysis of CoP displacement parameters via the Ramer-Douglas-Peucker algorithm **(A)** in one healthy participant **(B)**.

Gait initiation (GI) is defined as the process that starts with the onset of movement following an initiation signal and ends at the toe-off of the stance foot. This process poses significant challenges for individuals with PD. It comprises two main phases: the postural phase and the locomotor phase (LOC), as depicted in Figure 1. The postural phase involves anticipatory postural adjustments (APA), which include APA1 and APA2 (Remelius et al., 2008). APA2 is further subdivided into APA2a and APA2b. APA1 initiates with the onset of movement, involving initial lateral and posterior shifts of the CoP toward the swing foot heel, and concludes as the swing foot begins to unload. APA2 follows, from the unloading to toe-off, primarily featuring a lateral CoP shift toward the stance foot, facilitated by hip strategy movements. APA2a starts immediately after the heel-off of the swing leg, ensuring a smooth transition in step progression, while APA2b continues throughout the movement, focusing on maintaining stability (Chung et al., 2008; Remelius et al., 2008; Delval et al., 2012; Cau et al., 2014). Absences or incompleteness of APAs are observed across all age groups in healthy individuals (Lu et al., 2017). In people with PD, a significant reduction in APAs preceding voluntary steps is noted, which likely contributes to the hypokinetic gait patterns commonly observed in this group (Rogers et al., 2011; Russo et al., 2022).

APAs are primarily evaluated using two metrics: amplitude and duration. Amplitude refers to the intensity of muscle activity required to counteract potential destabilization from impending movements. Greater amplitudes suggest a more robust preparatory response, which is essential for maintaining postural integrity. Duration, in contrast, measures the time these muscle adjustments are sustained prior to the commencement of movement. An extended duration may indicate a delayed neuromuscular response, often reflective of coordination challenges. This assessment is especially critical in diagnosing and managing balance disorders in individuals with neurological conditions, where prolonged durations may signify impaired muscle coordination (Remelius et al., 2008). For people with PD, the amplitude and duration of APAs tend to show more variability, illustrating differences in force generation, duration, and the displacement of CoP before the first step (Jacobs et al., 2009).

Within the context of PD, research into how the severity of clinical symptoms correlates with the occurrence of falls is divided into two main areas: the use of clinical rating scales and quantitative biomechanical assessments of APAs. These assessments include force plate analysis (Cau et al., 2014; Onuma et al., 2021), electromyography of the leg muscles (Crenna and Frigo, 1991; Schlenstedt et al., 2018), accelerometers (Mancini et al., 2009; Onuma et al., 2021) and wearable technology. Additionally, specialized clinical scoring systems like the Tinetti balance and gait scores are utilized to assess the likelihood of falls among individuals with PD who do not have significant cognitive impairments (Kerr et al., 2010).

Our recent investigation has confirmed the effectiveness of using the CoP trajectory as a metric for APA in individuals experiencing freezing of gait (FOG) (Kung et al., 2023). However, the specific subphase within the APA stage that may critically influence fall risk remains unclear. Historically, the relationship between clinical disease severity and APA has been seen as a complicating factor in gait studies related to PD (Tard et al., 2013; Lira et al., 2020; Russo and Vannozzi, 2021). In contrast, the primary goal of mediation analysis is to identify causal mechanisms. This method helps explore not only the causal links between clinical disease severity and fall risk but also the extent to which APA duration impacts fall risk (Lai et al., 2022). Our study tests the hypothesis that the duration of the APA2 subphase serves as a mediator in the relationship between clinical disease severity and fall risk in people with PD. Additionally, we included a control group of healthy volunteers to establish a baseline or ‘normal’ reference for CoP displacement parameters, allowing for comparisons with the effects observed in the experimental group. According to our hypotheses, increased severity in clinical conditions is associated with prolonged APA duration. This extended duration may indicate compromised balance control during gait initiation, potentially leading to a higher risk of falls.

## Patients and methods

### Study design and patient selection

This study represents a secondary analysis of data from a prospective study (Kung et al., 2023) where we evaluated patients meeting the clinical diagnostic criteria for idiopathic PD as defined by the International Parkinson and Movement Disorder Society (Hughes et al., 1992; Postuma et al., 2015; Heim et al., 2017). Participants were recruited from an outpatient clinic, with inclusion criteria including steady daily anti-Parkinsonian medication and Hoehn and Yahr stage 1–3 for independent walking. Exclusion criteria were advanced PD stage (≥4), cognitive impairment (Clinical Dementia Rating (CDR) more than or equal to 1), balance-affecting etiologies, and lower limb weakness and dependence on assistance for walking. The study obtained approval from the hospital’s Institutional Review Committee on Human Research (IRB 201901802B0).

Sample size determination was conducted using G*Power software version 3.1.9.2, developed by the Universität Düsseldorf, Germany (Kang, 2021). A predetermined level of significance, denoted by *α* = 0.05, was established alongside one-tailed statistical tests. In the pursuit of statistical power, a value of 1–β was set at 0.8, and the effect size was specified as 0.13, with five independent variables. Ultimately, the study included 110 participants. All participants provided written informed consent after receiving detailed verbal and written information about the study. To facilitate clinical comparisons, 30 healthy volunteers matched for age and sex were recruited for the control group.

### Clinical assessment

Clinical evaluations, including assessments using rating scales, were conducted on patients in their off-medication states after at least 12 h of overnight fasting from dopaminergic medications. Data collected included patients’ age, disease duration, sex, body mass index (BMI), and levodopa equivalent daily dose (LEDD) (Tomlinson et al., 2010), along with a comprehensive medical history. The Unified Parkinson’s Disease Rating Scale (UPDRS) and Hoehn and Yahr stages were utilized to assess the clinical severity (Hoehn and Yahr, 1967; Martinez-Martin et al., 1994). Cognitive outcomes were assessed using the CDR scale, which measures functional capacity irrespective of physical disability. Additionally, the Cognitive Abilities Screening Instrument (CASI C-2.0), which includes 20 items across 9 domains, was used, with higher scores indicating better cognitive function (Chen et al., 2017). The Tinetti balance and gait scores were also used to assess fall risk in Individuals with PD, featuring a balance score from 0 to 16 points and a gait score from 0 to 12 points (Kerr et al., 2010).

### Measurement of GI parameters obtained by the CoP trajectory

CoP displacement trajectories were obtained using a MatScan pressure mat (Tekscan Inc., Norwood, MA, USA) model 3,150. The CoP displacement trajectory for gait initiation (GI) was analyzed to differentiate between the postural adjustment phases (APA1, APA2a, and APA2b) and the locomotor phase (LOC) (Remelius et al., 2008; Delval et al., 2012).

In our study, we chose to start a step and then stop, rather than walking continuously at the preferred speed, due to economic and space constraints. At the beginning of each trial, participants were instructed to stand barefoot on the MatScan, pressure mat (Tekscan Inc., Norwood, MA, USA), adopting a relaxed stance with feet parallel and heels aligned to pelvic width, maintaining this fixed position for 30 s. Upon receiving the verbal command ‘GO,’ participants were directed to initiate the first step with the most affected leg (for individuals with Parkinson’s Disease) or the right leg (for healthy volunteers) (Russo and Vannozzi, 2021), followed by stepping with the opposite leg. After reaching the new position with both feet parallel to the floor, participants were instructed to maintain a steady standing posture until the verbal command ‘STOP’ was issued. Data acquisition of the CoP displacement commenced three seconds prior to the ‘GO’ command to ensure an adequate baseline observation window and concluded after the verbal command ‘STOP’ was issued. Each participant, both individuals with PD in their off-medication state and healthy volunteers, underwent three successive trials. The mean values from these three consecutive recordings were used for subsequent analysis. If FOG persisting beyond the standard testing duration in any trial, that data would be excluded from analysis or the test would be repeated to ensure accuracy.

The Ramer-Douglas-Peucker algorithm was employed to simplify the CoP displacement trajectories and identify turning points (Guedri et al., 2021; Figure 1). We calculated the duration, length, and velocity of the CoP trace during the APA1, APA2a, APA2b, and LOC phases. Professionals evaluated and, if necessary, adjusted the identified turning points. We also compared the duration, length, and velocity of the CoP traces in the anteroposterior and mediolateral directions between PD patients and healthy volunteers. Data acquisition and analysis were conducted using Python, ensuring rigorous statistical assessment.

### Statistical analysis

**Figure 2:**
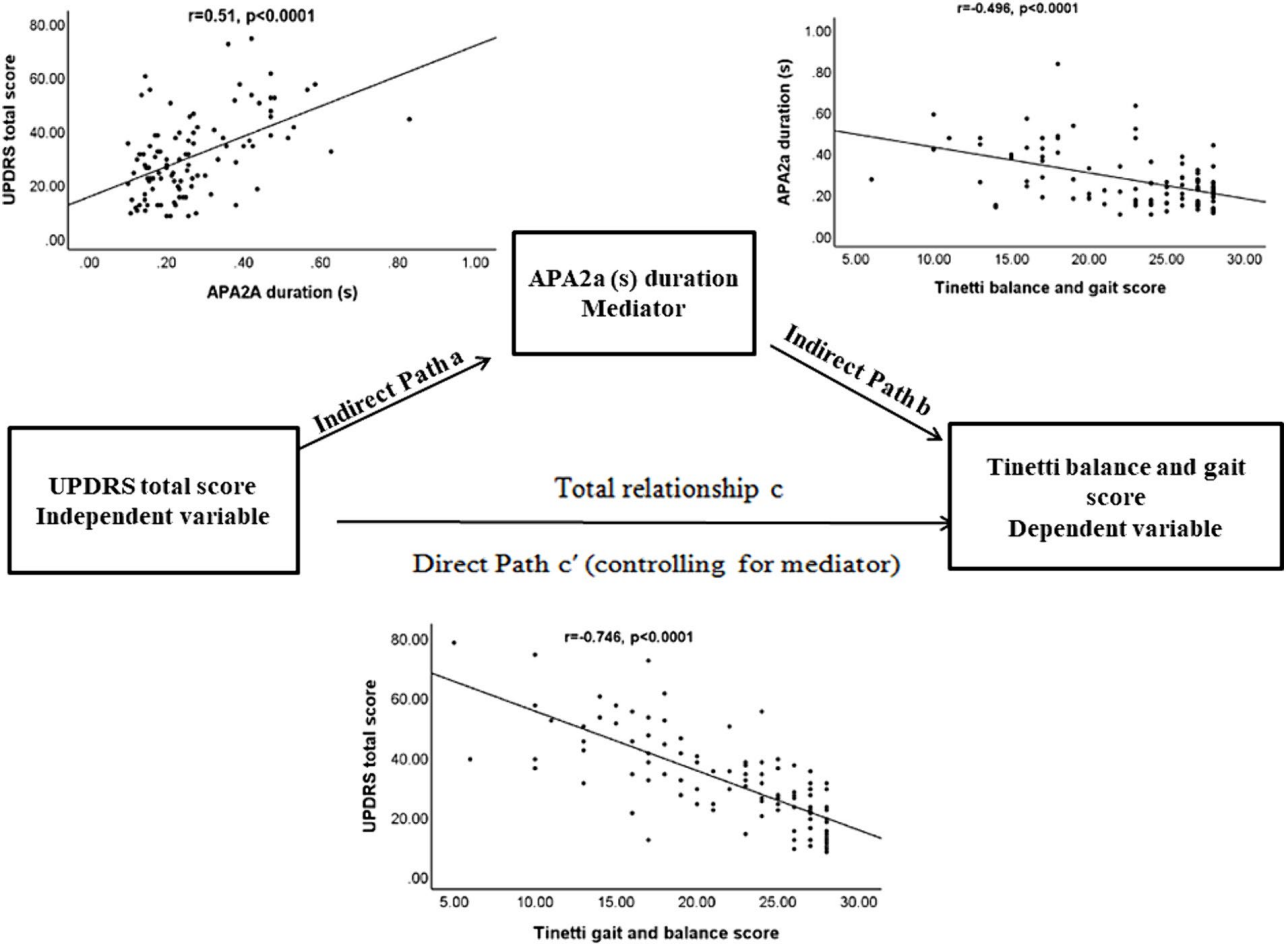
The diagram of the mediation hypothesis framework.

The data were expressed as mean ± standard deviation (SD). Baseline continuous variables between two groups (Individuals with PD and healthy volunteers) were compared using an independent t-test. Correlation analysis was conducted to explore the relationship between parameters of CoP displacement in different sub-phases of GI and UPDRS total score on Tinetti balance and gait score. Additionally, a stepwise multiple linear regression analysis was conducted to evaluate the influence of independent variables on the dependent variable (Tinetti gait and balance score). Furthermore, due to the noteworthy correlation detected among explanatory variables derived from CoP trajectories in our investigation, the identification of independent risk factors was conducted utilizing the least absolute shrinkage and selection operator (LASSO) regression approach. Finally, a mediation analysis was performed based on the linear regression results to determine whether the mediating variable (APA duration) could explain the relationship between the independent variable (UPDRS total score) and the dependent variable (Tinetti gait and balance score) (Figure 2). In our investigation, the UPDRS total score is employed as a validated metric for quantifying clinical disease severity (Lai et al., 2022). Additionally, the Tinetti gait and balance assessment, integrating balance and gait scores, is utilized in clinical practice to assess fall risk in Parkinson’s patients (Kerr et al., 2010). The statistical significance threshold was set at 0.05 for all relevant paths (Wager et al., 2008), and IBM SPSS Statistics v23 software (IBM, Redmond, WA, USA) was used for all statistical analyses.

## Results

### Clinical characteristics of patients

A total of 110 individuals with PD (mean age, 68.4 ± 9.48 years) and 30 healthy volunteers (mean age, 67.5 ± 9.18 years) participated in this study. The baseline characteristics of all participants are presented in Table 1.

**Table 1:** Baseline characteristics of patients.

	Patients (*n* = 110)
Age, years	68.1 ± 9.7
Sex (men/women)	55/55
Body mass index (kg/m^2^)	25.1 ± 4.2
Disease duration, years	5.3 ± 4.5
UPDRS total score (off-state)	31.1 ± 15.1
UPDRS I (Mentation, behavior, and mood)	1.7 ± 1.4
UPDRS II (ADL score)	9.7 ± 5.4
UPDRS III (motor score)	17.8 ± 9.5
UPDRS IV (motor complications)	1.7 ± 1.5
Hoehn and Yahr Staging	2.1 ± 0.9
LEDD (mg/day)	809.2 ± 571.8
Tinetti balance and gait score (off-state)	22.3 ± 5.7

UPDRS, Unified Parkinson’s Disease Rating Scale; ADL, activity of daily living; LEDD, Levodopa equivalent dose.

### Parameters on CoP displacement between individuals with PD during off-state and healthy volunteers

FOG was observed in 18 out of 110 participants during the measurement of CoP trajectories. Specifically, FOG occurred in all three trials for 4 participants, in two out of three trials for 7 participants, and in one out of three trials for 7 participants. The Ramer-Douglas-Peucker algorithm successfully identified CoP displacement trajectories in 105 participants (95.5%), while the remaining 5 cases could not be identified due to FOG. Among the 105 participants with identified trajectories, 8 participants exhibited one identifiable CoP displacement trajectory across the three trials, 7 participants exhibited two identifiable trajectories, and 90 participants exhibited identifiable CoP displacement trajectories in all three trials. Similarly, the Douglas-Peucker algorithm proficiently identified CoP displacement trajectories in all 30 healthy volunteers (100% success rate). Among these volunteers, 4 participants displayed a solitary identifiable CoP displacement trajectory throughout the three trials, 4 participants showcased two discernible trajectories, and 22 participants demonstrated consistent identification of CoP displacement trajectories across all three trials. Table 2 shows the duration, length, and velocity of baseline APAs between two groups: individuals with PD during off-state, and healthy volunteers. Individuals with PD had longer APA2a, APA2b duration and LOC (s) (APA2a [s], *p* = 0.003, APA2b [s], *p* = 0.005, and LOC (s), *p* = 0.003), shorter APA1, APA2a and LOC length (APA1[cm], *p* < 0.0001, APA2a [cm], *p* < 0.0001, and LOC [cm], *p* = 0.014), and lower velocity in mediolateral (APA1 [X] [cm/s], *p* < 0.0001, APA2a [X] [cm/s], *p* = 0.001, APA2b [X] [cm/s], *p* = 0.002, and LOC [X] [cm/s], *p* < 0.0001) and anteroposterior directions (APA1 [Z] [cm/s], *p* = 0.001, APA2a [Z] [cm/s], *p* < 0.0001, and APA2b [Z] [cm/s], *p* = 0.009) compared to healthy volunteers.

**Table 2:** Parameters on CoP displacement in patients with Parkinson’s disease and healthy volunteers.

	PD (*n* = 110)	healthy volunteers (*n* = 30)	*p*-value
APAs duration (s)			
APA1(s)	0.56 (0.24)	0.55 (0.18)	0.23
APA2a (s)	0.31 (0.16)	0.20 (0.08)	0.003
APA2b (s)	0.18 (0.07)	0.14 (0.04)	0.005
LOC (s)	0.78 (0.18)	0.67 (0.13)	0.003
APAs length (m)			
APA1 (cm)	2.62 (1.27)	4.92 (1.49)	<0.0001
APA2a (cm)	5.18 (2.02)	6.97 (2.69)	<0.0001
APA2b (cm)	5.62 (1.68)	6.24 (1.88)	0.90
LOC (cm)	14.55 (1.89)	15.51 (1.75)	0.014
APAs velocity (m/s)			
Mediolateral direction (X)			
APA1 (X) (cm/s)	3.64 (2.19)	6.64 (3.3)	<0.0001
APA2a (X) (cm/s)	10.03 (4.55)	16.1 (6.49)	0.015
APA2b (X) (cm/s)	9.36 (5.65)	12.94 (8.52)	0.96
LOC (X) (cm/s)	19.51 (5.49)	23.71 (5.66)	<0.0001
Anteroposterior direction (Z)			
APA1 (Z) (cm/s)	4.23 (2.38)	6.78 (3.67)	0.001
APA2a (Z) (cm/s)	16.75 (10.17)	32.19 (13.46)	<0.0001
APA2b (Z) (cm/s)	33.62 (11.31)	46.66 (15.07)	0.009
LOC (Z) (cm/s)	2.90 (2.0)	2.84 (2.39)	0.89

z = Track velocity (m/s) in the segments in the anteroposterior direction; x = Track velocity (m/s) in the segments and in the mediolateral direction.

### Correlation analysis between gait initiation parameters on CoP displacement and the UPDRS total score on Tinetti balance and gait score

The present study utilized correlation analyses to examine the effects of GI parameters on CoP displacement in the off state, levodopa equivalent daily dose, UPDRS total score, disease duration and Tinetti balance and gait score (Table 3). Our findings reveal that Tinetti balance and gait score exhibited a negative correlation with UPDRS total score (*r* = −0.746, *p* < 0.0001), LEDD (*r* = −0.402, *p* < 0.0001), as well as with various APA and LOC parameters, including APA2a (s), APA2b (s), and LOC (s) (*r* = −0.448, *p* < 0.0001, *r* = −0.304, *p* = 0.002 and *r* = −0.402, *p* < 0.0001), APA1 (cm) in APAs length (cm) (*r* = 0.315, *p* = 0.001), APA1 (X) (cm/s), APA2a (X) (cm/s), APA2b (X) (cm/s) in APAs and LOC (X) (cm/s) velocity (cm/s) in the mediolateral direction (X) (*r* = 0.294, *p* = 0.003, *r* = 0.325, *p* = 0.001, *r* = 0.246, *p* = 0.013 and *r* = 0.37, *p* < 0.0001), as well as APA2a (Z) (cm/s) and APA2b (Z) (cm/s) in APAs velocity (cm/s) in the anteroposterior direction (Z) (*r* = 0.252, *p* = 0.011 and *r* = 0.335, *p* = 0.001).

**Table 3:** Correlation analysis between parameters on CoP displacement, levodopa equivalent daily dose, and UPDRS total score on Tinetti balance and gait score.

Spearman correlation	Tinetti balance and gait score		
	*r*	*p*-value	Adjusted *p*-value^†^
Age	−0.11	0.068	0.086
Disease duration (years)	−0.05	0.024	0.035
Levodopa equivalent daily dose (mg/day)	−0.402	<0.0001	0.001
UPDRS total score	−0.746	<0.0001	0.001
APA parameters on CoP displacement			
APAs duration (s)			
APA1 (s)	0.11	0.26	0.297
APA2a (s)	−0.448	<0.0001	0.001
APA2b (s)	−0.304	0.002	0.005
LOC (s)	−4.02	<0.0001	0.001
APAs length (cm)			
APA1 (cm)	0.315	0.001	0.004
APA2a (cm)	−0.05	0.61	0.61
APA2b (cm)	0.18	0.07	0.086
LOC (cm)	0.19	0.06	0.078
APAs velocity (cm/s)			
Mediolateral direction (X)			
APA1 (X) (cm/s)	0.294	0.003	0.006
APA2a (X) (cm/s)	0.325	0.001	0.003
APA2b (X) (cm/s)	0.246	0.013	0.021
LOC (X) (cm/s)	0.37	<0.0001	0.001
Anteroposterior direction (Z)			
APA1 (Z) (cm/s)	0.10	0.33	0.35
APA2a (Z) (cm/s)	0.252	0.011	0.02
APA2b (Z) (cm/s)	0.335	0.001	0.003
LOC (Z) (cm/s)	0.11	0.29	0.32

UPDRS, Unified Parkinson’s Disease Rating Scale; z = Track velocity (m/s) in the segments in the anteroposterior direction; x = Track velocity (m/s) in the segments and in the mediolateral direction; † = Adjusted *p*-value is calculated by Benjamini-Hochberg false discovery rate.

### Significant clinical factors associated with Tinetti balance and gait score

A multiple linear regression analysis was performed to determine the significant variables influencing the augmented Tinetti balance and gait score in individuals with PD (Table 4). The regression model used the significant parameters from the correlation analysis in Table 3. The analysis revealed that UPDRS total score (*β* = −0.219, *p* < 0.0001) and APA2a (s) (*β* = −7.186, *p* = 0.024) were significant predictors of the Tinetti balance and gait score and all variance inflation factors (VIF) were < 5.

**Table 4:** Effects of the variables on Tinetti balance and gait score according to correlation analysis.

Univariable^α^	Model				
	Regression coefficient	Standard error	r_partial_	VIF	*p* value
Constant	31.29	0.932			<0.0001
UPDRS total score (off-state)	−0.219	0.029	−0.640	1.352	<0.0001
APA2a (s)	−7.186	3.132	−0.211	1.352	0.024

α = Significant univariable included LEDD, UPDRS total score, disease duration, APA2a (s), APA1 (m), APA2b (m), APA1 (X) (m/s), APA2a (X) (m/s), APA2b (X) (m/s), APA2a (Z) (m/s), APA2b (Z) (m/s), LOC (s) and LOC (X) (m/s). APA, Anticipatory postural adjustment; LEDD, levodopa equivalent daily dose; UPDRS, Unified Parkinson’s Disease Rating Scale; VIF, variance inflation factor. Model: R^2^ = 0.54. Predictors in the model: constant, UPDRS total score (off-state), APA2a (s).

### Mediation analysis of clinical disease severity, APA duration, and fall risk

The primary hypothesis of this analysis is to investigate the possibility of an indirect relationship between clinical disease severity (as the independent variable measured by UPDRS total score) and the risk of falling (as the dependent variable assessed by Tinetti balance and gait score) through the mediation of APA duration, while controlling for significant group main effects. A path model was used to test three effects: (a) UPDRS total score on mediator (APA duration), (b) mediator on dependent variable (Tinetti score) while accounting for APA duration, and (c) mediation effect. To simplify, we report a comprehensive list of the results from the present study that fulfill the three criteria mentioned above. All the mediation relationships were found to be statistically significant (*p* = 0.047, Sobel test) (Figure 2; Table 5).

**Table 5:** A simple mediation model of Clinical diseases severity (UPDRS total score [X]) on the Risk of falling (Tinetti balance and gait score [Y]) through APA Duration (APA2a (s) [M]) effort.

	Path coefficient	Standard error	*p*-value
Total effects (total relationship, path c)^Ω^			
The relationship between the UPDRS total score and Tinetti balance and gait score	−0.279	0.024	<0.0001
Direct effects, path c′			
The relationship between the UPDRS total score and Tinetti balance and gait score by including the APA2a (s) into the model	−0.233	0.028	<0.0001
Indirect effect, path a			
The effect of the UPDRS total score on the APA2a (s)	0.005	0.001	<0.0001
Indirect effect, path b			
The effect of the APA2a (s) on the Tinetti balance and gait score	−19.76	3.428	<0.0001

X = UPDRS total score (independent variable); Y = Tinetti balance and gait score (dependent variable); M = APA2a value (mediator). Ω = The mediation effects a × b which is defined as the reduction of the relationship between the independent and dependent variables (Tinetti balance and gait score-UPDRS total score) (total relationship, path c) by including the mediator into the model (direct path, path c′) (Sobel test, *p* = 0.047).

## Discussion

### Major findings

Our study’s findings are in line with our hypothesis and are consistent with existing literatures (Tard et al., 2013; Lira et al., 2020; Russo and Vannozzi, 2021), showing that individuals with PD with more severe symptoms exhibited higher UPDRS total scores, extended APA duration, and reduced Tinetti balance and gait scores. Our results highlight the relationship between UPDRS total score, APA duration, and the risk of falls in individuals with PD.

### The pathogenesis of APAs in gait initiation difficulties and its correlation with PD severity

APAs have been thoroughly investigated in individuals with PD due to their pronounced balance deficits, challenges with gait, and high incidence of falls (Mirelman et al., 2019; Russo et al., 2022). Research by Jacobs et al. highlights the pivotal role of the supplementary motor area (SMA) in motor control within this context. Their study, employing repetitive transcranial magnetic stimulation to selectively disrupt SMA function, demonstrated that such disruption leads to significant changes in the amplitude and duration of APAs in individuals with PD, as compared with control subjects (Jacobs et al., 2009). Furthermore, Chastain et al. showed that stimulation of the subthalamic nucleus (STN) improves both anteroposterior and vertical parameters of gait initiation, suggesting that gait initiation, which likely reflects postural control during gait, is more dependent on the activity of the basal ganglia, especially the STN, rather than directly on the brain’s dopaminergic systems (Chastan et al., 2009). Additionally, Lencioni et al. found that APA parameters, when assessed using the MDS-UPDRS III score, were strongly correlated with the severity of PD, particularly impacting balance stability and gait (Lencioni et al., 2022). These findings enhance our understanding of how different brain regions, particularly the SMA and STN, contribute to APA in GI for Individuals with PD, underlining the complex interactions that regulate motor functions and postural stability.

### The significance of APA2 subphase relative to APA1 in PD with implications for falls risk

Individuals with PD is associated with increased duration of APAs, and decreased length and velocity of APAs in the mediolateral (X) and anteroposterior (Z) directions, which may explain difficulties in GI (Jacobs et al., 2009; Mancini et al., 2009). In a prior investigation (Remelius et al., 2008), it was observed that longer durations in GI predominantly stemmed from extended durations in APA2 and double support phases with relatively shorter durations in APA1 in multiple sclerosis participants. In the other study (Cau et al., 2014), it was discovered that obese participants exhibited heightened medio-lateral movement, resulting in statistically significant increments in both the length and duration of their APA2a phase. Evaluating the coordination and interplay between APA2 and APA1 in individuals with PD can offer critical insights into gait impairments, balance deficits, and the risk of falls. The recent extensive cohort study on PD (De Waele et al., 2024) revealed that individuals with PD experience prolonged GI time after a verbal cue and exhibit increased durations of APA1 and APA2a compared to healthy controls. The extended duration of APA2a implies diminished control during GI, consequently elevating the risk of falls.

### Rationale for the preference of APA duration as a mediating variable over APA amplitude

The assessment of APAs involves two primary metrics: amplitude and duration. The amplitude of APAs can be computed by assessing the displacement of the CoP in both the anteroposterior (AP) and mediolateral (ML) directions during the preparatory phase of GI (Huang and Brown, 2013). APA duration, measured from CoP displacement onset to reaching movement initiation, signifies the temporal extent of muscular adjustments preceding movement. This parameter is critical for detecting coordination deficits, especially in neurological conditions where prolonged APA durations may indicate compromised neuromuscular coordination and increased fall risk during GI (e.g., FOG). Studies, including those exploring interventions like repetitive transcranial magnetic stimulation in individuals with PD, suggest that changes in APA duration may exhibit greater sensitivity to intervention effects compared to APA amplitude (Jacobs et al., 2009). In summary of the preceding discussion, the duration of APA2a could potentially serve as a critical parameter within the APA stage, exerting a pivotal influence on the genesis of fall risk.

### Identification of candidate variables for mediation analysis: a regression-based approach

Given the notable correlation observed among CoP-based explanatory variables, our approach was to exclusively incorporate variables showing significant correlations with Tinetti balance and gait scores, as detailed in Table 3, into our stepwise multiple linear regression analysis models. Employing stepwise procedures and evaluating collinearity statistics, our statistical analysis revealed robust associations between the UPDRS total score and APA2 duration with Tinetti balance and gait scores. It is noteworthy that all VIF remained below the threshold of 5. These VIF values, falling within the range of 1 to 5, indicate a moderate level of correlation, thereby not necessitating corrective interventions. In the conclusive stepwise linear regression analysis, it was observed that solely APA2a (s) and UPDRS total score emerged as statistically significant predictors of the Tinetti balance and gait score. Moreover, the predictive factors were subjected to LASSO regression analysis, renowned for its efficacy in addressing multicollinearity challenges inherent in variable sets. Consequently, two variables exhibiting discernible non-zero coefficients were ascertained, specifically APA2a (s) and UPDRS total score, thus emerging as potential predictors. Following this identification, APA2a duration (s), UPDRS total score, and Tinetti balance and gait scores were delineated and subsequently selected as candidate variables for mediation analysis.

### Rehabilitation programs focusing on anticipatory postural adjustments (APA2a)

APA2 involves a lateral shift of the CoP toward the stance foot from unloading to toe-off, facilitated by hip movements. APA2a starts immediately after the swing leg’s heel-off, ensuring smooth step progression. Rehabilitation programs focusing on APA2a are crucial for improving gait and mobility in PD. Key components include postural control exercises for core stability and balance, gait training to enhance posture and weight transfer, coordination drills for motor planning, and feedback to increase APA2a awareness (Domingos et al., 2018). These elements improve postural control, gait function, and overall mobility, reducing fall risk and enhancing functional outcomes for individuals with PD.

### Clinical implications and outline future research priorities

A notable strength of our study is the validation of APA2 duration as a mediator in the causal mechanisms linking clinical disease severity and fall risk in PD, using CoP displacement trajectories during GI. The duration of APA2a may serve as a crucial parameter within the APA stage, significantly influencing the development of fall risk. Future longitudinal studies are essential to track changes in APA characteristics over time and to correlate these changes with the progression of PD. Such studies will help determine whether APA changes can predict worsening motor symptoms and increased risk of falls, thereby providing potential biomarkers for disease progression. Additionally, exploring how different pharmacological treatments for PD affect APA and their efficacy in reducing fall risk could further elucidate the pathophysiological interactions between PD pharmacological effects and motor control. Moreover, nonpharmacological approaches, including customized physical therapy regimens focused on enhancing motor control during GI and alternative therapies, show promise in improving balance and motor function in PD. Neurostimulation techniques, particularly those targeting the function of brain regions involved in APA regulation like the SMA and STN, should also be considered. These approaches could provide comprehensive insights into the symptoms of the disease and lead to more effective treatments for PD.

## Limitations

Our study identifies five limitations. The first concerns our methodology related to the lateral displacement of CoP. Although participants were instructed to adopt a standardized stance with heels aligned to pelvic width, we did not normalize the lateral displacement of the CoP for stance width variations. This oversight may affect the interpretation of APAs and their correlation with balance control. Future studies should consider standardizing or normalizing stance width to ensure more precise APA assessments. Secondly, by excluding individuals with advanced PD or mild-to-moderate dementia, our study is limited in its ability to evaluate the impact of CoP displacement measurements on a broader spectrum of individuals with PD, particularly those at higher risk of falls. Thirdly, our results provide only background neurological status, and we did not record our patients during the on-medication state. Fourthly, the margin of stability (MoS), a critical metric for assessing dynamic gait stability, was not measured in our study. MoS, calculated by analyzing the difference between the base of support (BoS) and extrapolated center of mass (XcoM), with positive values indicating stability (XcoM within BoS) and negative values suggesting instability (XcoM outside BoS), provides essential insights into balance control. Although not included in our current research, incorporating MoS in future studies could significantly enhance our understanding of gait stability, especially in individuals with compromised stability. Fifthly, the presence of FOG is recognized as a significant predictor of fall risk, which can complicate evaluations of clinical disease severity. Lastly, we employed the Ramer-Douglas-Peucker algorithm for identifying APA phases, which is generally more robust than other methods. This algorithm detects turning points in CoP displacement trajectories, enabling the identification of APA components. If APA components are not initially produced, the algorithm cannot detect them and may misinterpret the trajectories. To mitigate this, future work will include collecting data from an outpatient clinic and developing app-embedded CoP trajectory tools to assist clinicians in decision-making.

## Conclusion

Our study underscores the critical role of APA2a duration as a mediator in the relationship between the severity of clinical disease and the risk of falls in individuals with PD. Continuous monitoring of APA measures, especially during the APA2a subphase, could form the foundation for developing targeted pharmacological and rehabilitation interventions. These interventions aim to reduce fall risk by addressing prolonged APA2a durations, as demonstrated by the outcomes of our research.

## Data availability statement

The raw data supporting the conclusions of this article will be made available by the authors, without undue reservation.

## Ethics statement

The studies involving humans were approved by Institutional Review Board of Chang Gung Medical Foundation (IRB 201901802B0 and 202201719B0). The studies were conducted in accordance with the local legislation and institutional requirements. Written informed consent for participation in this study was provided by the participants’ legal guardians/next of kin.

## Author contributions

C-HH: Data curation, Investigation, Writing – original draft. Y-RL: Data curation, Investigation, Writing – original draft. C-CH: Data curation, Formal analysis, Investigation, Writing – original draft. C-YL: Data curation, Investigation, Writing – review & editing. Y-SC: Data curation, Investigation, Writing – review & editing. C-CY: Data curation, Investigation, Writing – review & editing. S-YL: Data curation, Investigation, Writing – review & editing. W-CL: Methodology, Supervision, Writing – review & editing. B-CC: Methodology, Supervision, Writing – review & editing. W-CC: Data curation, Investigation, Writing – review & editing. Y-FC: Data curation, Investigation, Writing – review & editing. C-FK: Software, Writing – review & editing. C-HL: Conceptualization, Data curation, Formal analysis, Funding acquisition, Investigation, Methodology, Project administration, Resources, Supervision, Validation, Visualization, Writing – review & editing.
